# Construction of a novel inflammatory-related prognostic signature of acute myelocytic leukemia based on conjoint analysis of single-cell and bulk RNA sequencing

**DOI:** 10.3389/fimmu.2025.1565954

**Published:** 2025-06-10

**Authors:** Yongfen Huang, Ping Yi, Yixuan Wang, Lingling Wang, Yongqin Cao, Jingbo Lu, Kun Fang, Yuexin Cheng, Yuqing Miao

**Affiliations:** ^1^ Department of Hematology, Yancheng No.1 People’s Hospital, Yancheng, China; ^2^ Department of Scientific Research Project, Wuhan Kindstar Medical Laboratory Co., Ltd., Wuhan, China; ^3^ Kindstar Global Precision Medicine Institute, Wuhan, China; ^4^ Yancheng Clinical College, Xuzhou Medical University, Yancheng, China

**Keywords:** acute myeloid leukemia, ScRNA-seq, bulk RNA-seq, prognostic signature, inflammation

## Abstract

**Introduction:**

The prognostic management of acute myeloid leukemia (AML) remains a challenge for clinicians. This study aims to construct a novel risk model for AML patient through comprehensive analysis of scRNA and bulk RNA data to optimize the precise treatment strategies for patients and improve prognosis.

**Methods and Results:**

scRNA-seq classified cells into nine clusters, including Bcells, erythrocyte, granulocyte-macrophage progenitor (GMP), hematopoietic stem cell progenitors (HSC/Prog), monocyte/macrophagocyte (Mono/Macro), myelocyte, neutrophils, plasma, and T/NK cells. Functional analysis demonstrated the important role of inflammation immune response in the pathogenesis of AML, and the leukocyte transendothelial migration and adhesion in the process of inflammation should be noticed. ssGSEA method identified four core cells including GMP, HSC/Prog, Mono/Macro, and myelocyte for subsequent analysis, which contains 1,594 marker genes. Furthermore, we identified AML-associated genes (2,067genes) and DEGs (1,010genes) between AML patients and controls usingGSE114868dataset. After performing intersection, univariate Cox, and LASSO analysis, we obtained a prognostic model based on the expression levels of five signature genes, namely, CALR, KDM1A, SUCNR1, TMEM220, and ADM. The prognostic model was then validated by two external datasets. Patients with high-risk scores are predisposed to experience poor overall survival. Further GSEA analysis of risk-model-related genes revealed the significant differences in inflammatory response between high-and low-risk groups.

**Conclusion:**

In conclusion, we constructed an inflammation related risk model using internal scRNA data and external bulk RNA data, which can accurately distinguish survival outcomes in AML patients.

## Introduction

Acute myeloid leukemia (AML) is a highly heterogeneous malignant clonal disease derived from myeloid hematopoietic stem cells and progenitor cells characterized by abnormal proliferation of blast cells and leukemia cells in the bone marrow, thus leading to hematopoietic dysfunction. It is the most common subtype of leukemia, with 20,800 new cases and 11,220 deaths in 2024 in the United States (1). In the past 5 decades, AML therapy primarily depended on one poorly tolerated and modestly effective standard of care: the cytarabine combined with anthracyclines (2). Despite the advances in strategies containing high-dose cytarabine, targeted therapy, allogeneic hematopoietic stem cell transplantation, and immunotherapy, its 5-year overall survival (OS) is only 30% and <10% in patients older than 60 years (2, 3). Therefore, it is of great significant to discover novel therapeutic target and prognostic signature to guide treatment, thereby improving outcome of patients.

Bulk transcriptome sequencing (bulk RNA-seq) is an effective tool to profile average gene expression in cell populations, identifying abnormal expressed genes as potential therapy targets for diseases, including AML. Li et al. identified METTL3 as a biomarker of AML chemoresistance, providing a novel target for AML therapy (4). In contrast to bulk RNA-seq, single-cell RNA-seq (scRNA-seq) reveals the cell-type heterogeneity and genetic information by elucidating the transcriptomic diversity among individual cells, beneficial to specify personalized therapeutic schedule and disease diagnosis (5, 6). As reported by Bijender Kumar, contact between AML blasts and NK cells activated TGF-β and, in turn, contributing to NK cell exhaustion. BATF was identified as a key transcription factor that mediates NK-cell dysfunction in AML, implying that the adoptive transfer of allogeneic healthy NK cells in combination with TGF-β inactivation or BATF suppression might be a promising method for AML immunotherapy (7). Tian et al. suggested ENO1 as a plausible candidate for AML therapy and prognostic assessment using scRNA-seq, due to its specific function in self-renewal of leukemia stem cell (8). Given these advantages, bulk RNA-seq and scRNA-seq are frequently employed in combination for patient stratification and therapy (9, 10).

In this work, we mapped the immune microenvironment landscape and determine how it contributes to the progression of AML using scRNA-seq. In addition, we conducted comprehensive bioinformatics analyses using scRNA-seq and bulk RNA-seq data to obtain a prognostic signature of AML patients, with two online validation sets to verify its reliability for risk stratification.

## Materials and methods

### Participants

A total of 11 patients diagnosed as AML were eligible in this study and divided into favorable (group 1), intermediate (group 2), and unfavorable (group 3) prognosis groups according to risk stratification. The baseline characteristics of included AML patients are shown in **Table 1**. Two matched individuals with monophyletic reduction were involved as control. Bone marrow (BM) of all included patients was obtained for scRNA-seq. Human experimental procedures in this study were reviewed and approved by the ethics committee of Yancheng No. 1 People’s Hospital, and all participants provide written informed consent.

**Table 1:** The baseline characteristics of 11 AML patients collected for single-cell sequencing.

Characteristic	AML patients
Age (median, range)	70 (60–87)
Sex (n, %)	
Male	6 (55%)
Female	5 (45%)
WBCs (×10 ^9^/L, median, range)	3.02 (0.66–53.94)
Cytogenetic risk (n, %)	
Favorable	1 (9%)
Intermediate	4 (36%)
Unfavorable	6 (55%)
Mutation (n, %)	
ASXL2	1 (9%)
ASXL1	2 (18%)
RUNX1	3 (27%)
DNMT3A	1 (9%)
TET2	2 (18%)
KIT	1 (9%)
IDH2	3 (27%)
IDH1	2 (18%)
Negative	1 (9%)
Missing	1 (9%)
Extramedullary infiltration (n, %)	
Yes	0
No	10 (100%)
Fusion gene (n, 10%)	
CBFβ-MYH11	1 (9%)
AML1-ETO	1 (9%)
EVI1	1 (9%)
Negative	7 (64%)
Missing	1 (9%)

### Single-cell library preparation, sequencing, and data analysis

The BM sample were first prepared as single-cell suspension at a concentration of 700–1,200 cell/μ, followed by single-cell capture, mRNA reverse transcription, cDNA amplification, and 3′ sc-RNA-seq library construction using the 10X Genomics platform according to the manufacturer’s instructions. After quality inspection, the library was sequenced using Illumina platform. The obtained data were quantified using cellranger software to generate cell–gene expression matrix, followed by quality control. The umap algorithm was employed for dimensionality reduction analysis. The “singleR” package in R software with HumanPrimaryCellAtlasData, BlueprintEncodeData, and ImmuneCellExpressionData as reference was used for cell annotation based on marker genes finding from the CellMarker database and previous studies. By using the FindAllMarkers function of Seurat package, the marker genes for each cell type were obtained, and significantly different marker genes of each cluster were identified according the metrics of: logFC > 1 and adj.P.Val < 0.05. Then, these differently expressed marker genes and ssGSEA were used to calculate ssGSEA scores of each cell to acquire the different cells between AML patients and control individuals. GO and KEGG enrichment analysis of differently expressed marker genes and GSEA enrichment of all marker genes of selected cells were performed using clusterProfiler package in R software. CellPhone DB v2.0 was used to explore the potential interactions between different cells.

### Bulk RNA-seq datasets acquisition and processing

Bulk RNA-seq data of GSE114868 were downloaded from GEO database, containing 194 AML patients and 20 healthy donors. The differently expressed genes (DEGs) between AML and healthy donors were filtered based on the categories of logFC > 1 and adj.P.Val < 0.05, and genes associated with AML were screened using the R package WGCNA to find out overlapped genes of scRNA-seq. GSE37642 dataset, including 417 AML patients, was downloaded from GEO database and termed as training set to construct the prognostic signature of AML patients based on the overlapped genes. TCGA_LAML dataset (containing 130 AML patients) downloaded from TCGA database and GSE106291 dataset (containing 250 AML patients) downloaded from GEO database were termed as validation set to validate the constructed prognostic signature.

### Prognostic signature construction and validation

The overlapped genes of differently expressed genes between AML and healthy donors in GSE114868 dataset, AML-linked genes in GSE114868 dataset, and selected marker genes of scRNA-seq were considered as candidates. Univariate Cox regression analysis was employed on these candidates in the training set to filtered out informative genes correlated with prognosis. Genes with p < 0.05 were included in the least absolute shrinkage and selection operator (LASSO) regression analysis to resolve the final variables in prognostic model. The risk model was constructed based on the formula: risk score = gene exp1 × β1 + gene exp2 × β2 + … + gene expression n × βn, where gene expression represents the gene expression value and β represents the corresponding coefficient of LASSO regression. Patients were classified into high- and low-risk groups according to the median value of risk score. Subsequent prognostic model validation was performed on external datasets TCGA_LAML and GSE106291. Kaplan–Meier curves visualized by “survminer” package was utilized to analyze prognostic value of the two groups and the final remaining genes. The risk map of patients was visualized using “ggrisk” package in R software. The difference in risk scores among available subtypes was determined using the Kruskal–Wallis test or the Wilcoxon rank test. The receiver operating characteristic (ROC) curves were plotted using the “survROC” package to evaluate the performance of risk scores in predicting overall survival at 1, 2, 3, and 4 years in AML patients. In addition, a stratified survival analysis of available clinicopathological characteristics was carried out for high- and low-risk groups. In addition, the functional enrichment of the risk model associated genes was predicated using GSEA.

### Immune microenvironment analysis and chemotherapy drug sensitivity analysis

Immune microenvironment-related scores containing ImmuneScore, StromalScore, and ESTIMATEScore were calculated per AML patients in TCGA-LAML dataset using corresponding algorithm. The difference in each score between high- and low-risk AML samples was determined using the Wilcoxon rank sum test, and Pearson correlation analysis was performed to evaluate the correlation between our constructed prognostic signature and immune microenvironment status. Furthermore, we predicted the sensitivity of 198 drugs for the high- and low-risk groups using the oncoPredict package in R software, and drugs that are statistically different and potentially useful for AML treatment were selected for correlation analysis with target gene.

### Statistical analysis

All statistical computations and graphical representations were executed in R statistical software. Inter-group comparisons were conducted using t-tests and Wilcoxon rank-sum tests. For multi-group analyses, the Kruskal–Wallis test was systematically applied. Survival outcomes were evaluated through Kaplan–Meier estimators with between-curve differences quantified by log-rank testing. Prognostic biomarkers were identified using univariate Cox regression model. Bivariate associations were examined through Spearman’s rank correlation analysis. All inferential analyses maintained a predetermined p level of 0.05 for statistical significance determination.

## Results

### Cell subtype identification of included individuals

**Figure 1:**
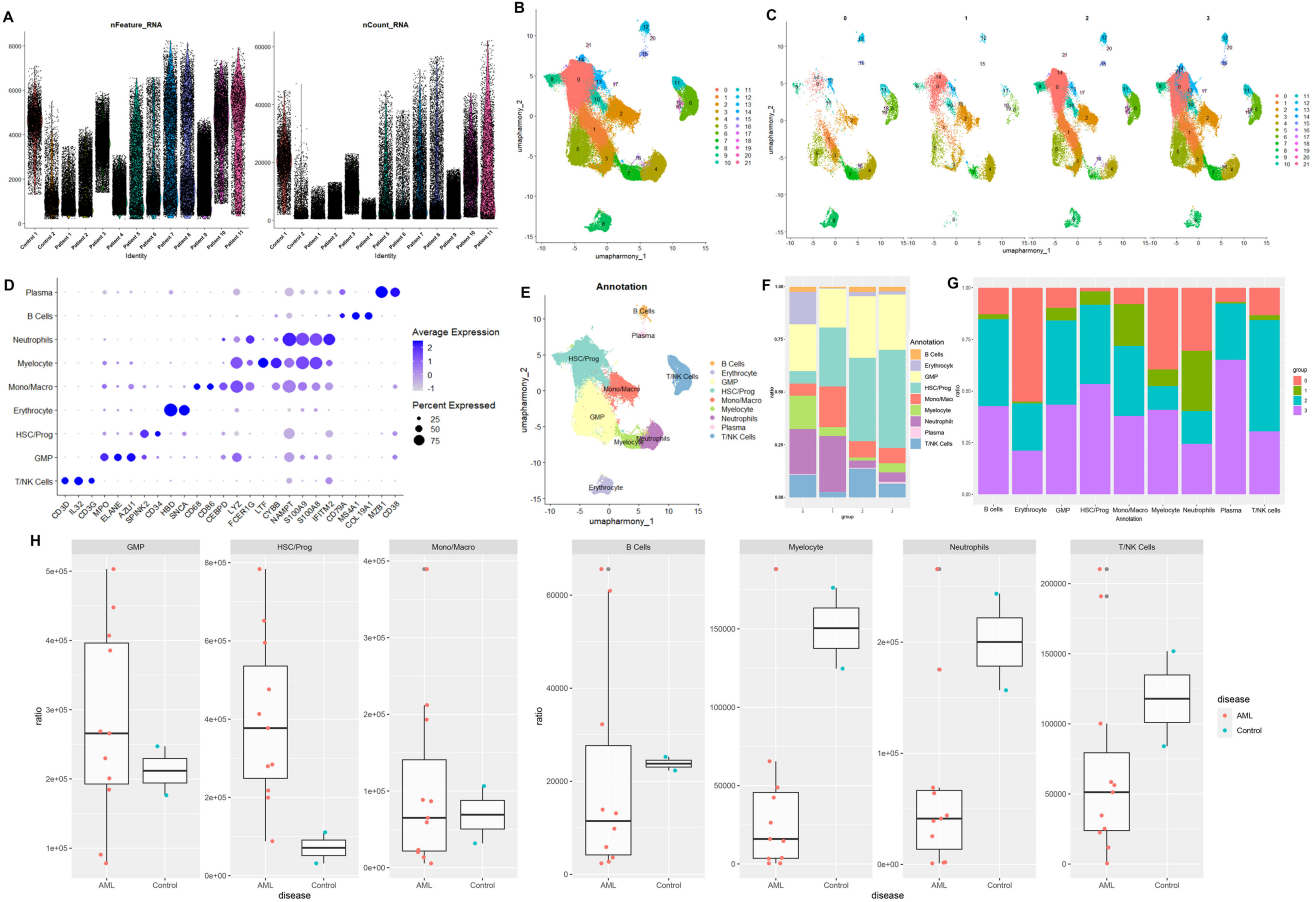
Quality control and cell type annotation of single-cell RNA sequencing. **(A)** Violin plot of filtered single-cell RNA sequencing data, showing a total of 97,531 cells meeting the criteria. **(B)** The umap algorithm was employed to classify the obtained cells, and 21 independent clusters were successfully classified. **(C)** Classification of cell clusters in each group. **(D)** Expression levels of cell marker genes in each cell cluster. **(E)** Cell-type annotation based on the corresponding marker genes, with nine cell clusters obtained. **(H)** The proportion of annotated cells in AML patients and healthy donors.

A total of 97,531 core cells were obtained for subsequent analysis after quality control (**Figure 1A**), and classified into 21 independent clusters using the umap algorithm (**Figures 1B, C**). Then, the various clusters were annotated using corresponding marker genes found in CellMarker database and previous references (**Figure 1D**), classifying into nine cell clusters, including B cells, erythrocyte, granulocyte-macrophage progenitor (GMP), hematopoietic stem cell progenitors (HSC/Prog), monocyte/macrophagocyte (Mono/Macro), myelocyte, neutrophils, plasma, and T/NK cells (**Figure 1E**). The different subtype demonstrated discrepant proportion in each group (**Figures 1F, G**), and the ratios of GMP, HSC/Prog, Mono/Macro, B cells, myelocyte, neutrophils, and T/NK cells in all cells are significantly different between AML patients and controls (**Figure 1H**), which were consistent with previous studies (11).

### Cell interaction and functional enrichment analysis of various cellular marker genes

**Figure 2:**
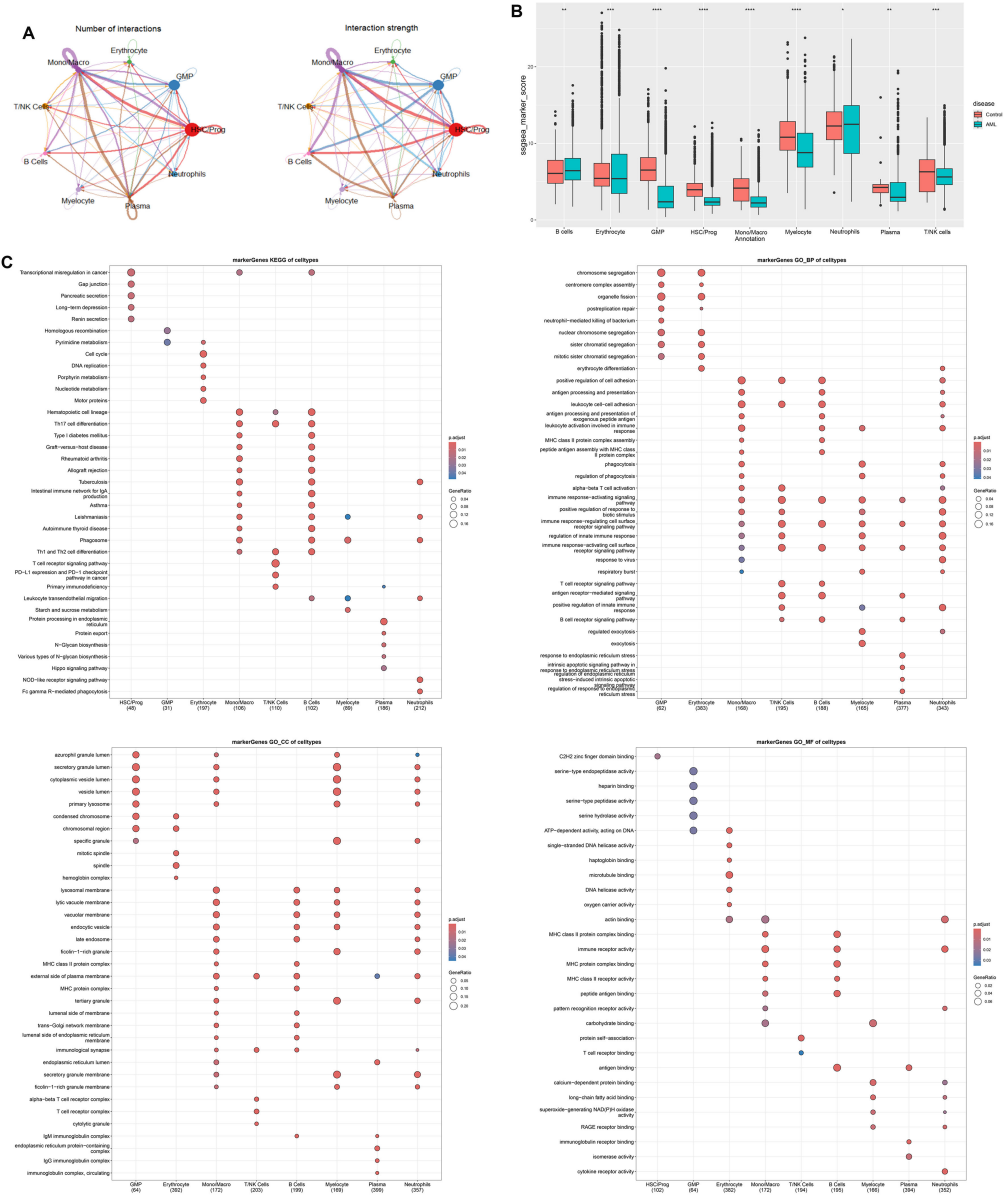
Cell–cell communication analysis and function enrichment of marker genes based on nine cells clusters. **(A)** Number and strength of interactions between major cells. **(B)** Differentially expressed cells in AML and controls were obtained by calculating the ssGSEA score of each cluster based on the marker genes. **(C)** KEGG and GO enrichment analysis based on marker genes of differentially expressed cells. *p < 0.05, ** p < 0.01, *** p < 0.001, **** p < 0.0001.

Intercellular interaction was predicted based on characteristic ligand receptors and pathways. As shown in cell–cell communication networks, there was more frequency and intensity of interaction between Mono/Macro and HSC/Prog, Mono/Macro and plasma, and Mono/Macro and GMP. T/NK cells demonstrated relatively poor communication with other cells (**Figure 2A**). Moreover, the significantly different marker genes of each cell subtype were identified using FindAllMarkers function of Seurat, followed by calculating ssGSEA scores. We found that the scores of all the identified cell clusters between AML patients and control individuals are significantly different, especially GMP, HSC/Prog, Mono/Macro, and myelocyte that are obviously downregulated in AML patients. Thus, these four cell types were regarded as core cells for subsequent analysis. In addition, the marker genes of these selected four clusters were enriched for KEGG and GO functions (**Figure 2C**). In detail, the marker genes for HSC/Prog, Mono/Macro, and B cells were associated with transcriptional misregulation in cancer. The marker genes of Mono/Macro, T/NK cells, and B cells were notably enriched in Th17 cell differentiation. In addition, the maker genes of B cells, myelocyte, and neutrophils were enriched in leukocyte transendothelial migration. In terms of GO enrichment, the marker genes of Mono/Macro, T/NK cells, B cells, myelocyte, and neutrophils were enriched in several immune-response-related terms at BP category.

### AML-associated key modules and DEG identification using bulk RNA-seq

**Figure 3:**
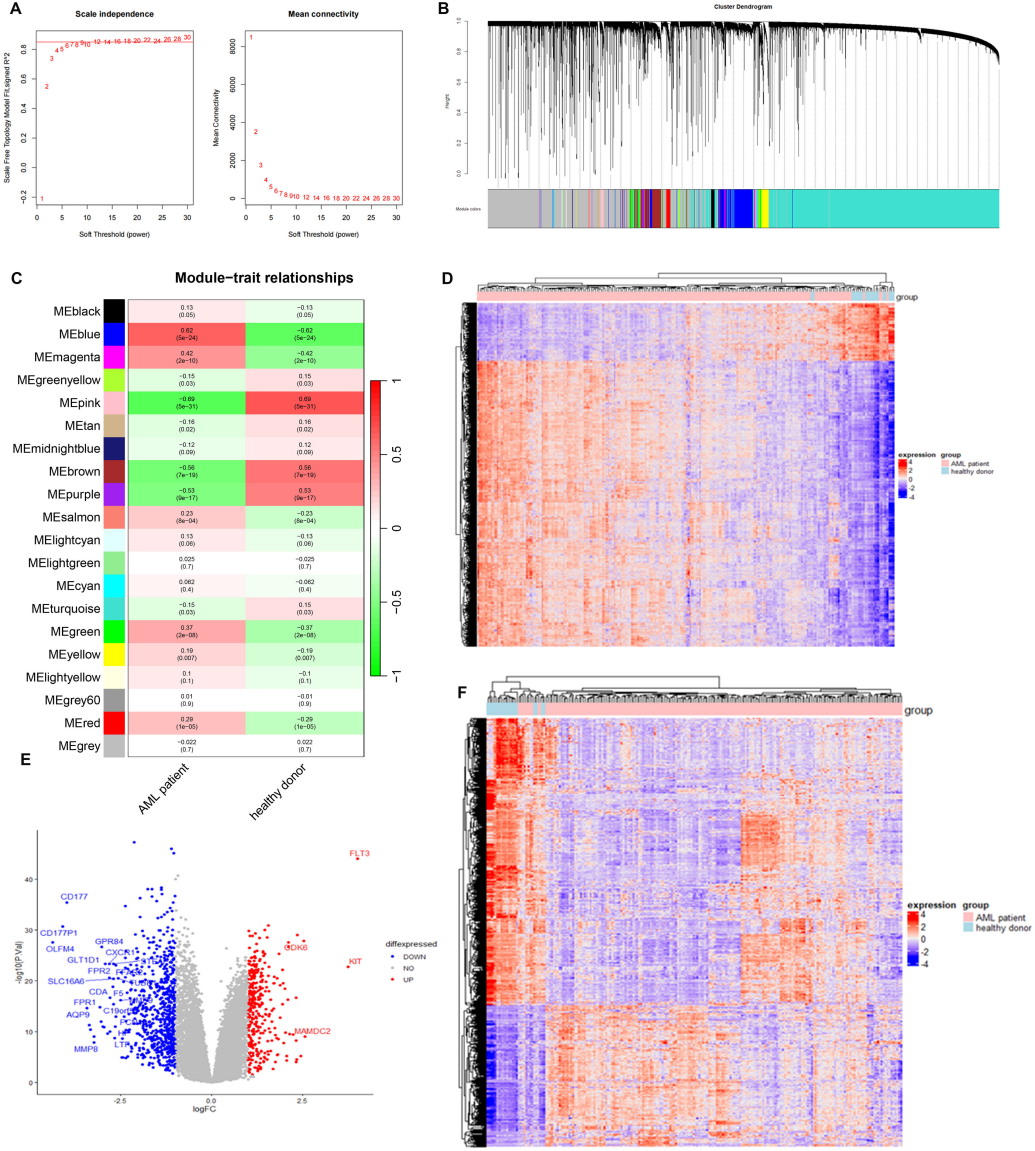
Identification of AML-associated genes and differential expressed genes in AMP patients using GSE114868 dataset. **(A)** Analysis of the scale-free index for various soft-threshold powers (β). **(B)** Gene cluster dendrogram and gene module colors. **(C)** Heatmap of correlations between modules and AML. **(D)** Heatmap of AML-associated genes in MEblue model. **(E)** The volcano plot of differentially expressed genes between AML patients and controls. Each blue dot represents a downregulated gene in AML patients, and each read dot represents an upregulated gene in AML patients. **(F)** Heatmap of differential expressed genes between AML patients and controls.

The GSE114868 dataset was downloaded to identify major modules associated with AML and DEGs between AML patients and controls. In details, WGCNA was employed to analyze genes associated with AML development and progression. The soft-thresholding power was optimized for the network topology by balancing the criteria of mean connectivity and scale-free topology fit, ensuring biologically interpretable network sparsity while preserving scale-independent properties. Setting the scale-free topology model fit index threshold to 0.85, we selected β = 7 as the soft threshold, at which the scale-free network performed optimally (**Figure 3A**). Highly similar modules were then clustered, and a total of 20 modules were available after dynamic hybrid cutting (**Figure 3B**). We then calculated the correlation between AML and the genes modules. Based on the p-value and correlation coefficient, we finally chose MEblue module (containing 2,967 genes) as the targeted module, which has the strongest positive association with AML (**Figure 3C**). The clustering of genes in MEblue module can separate AML patients from healthy donors properly (**Figure 3D**). A total of 1,010 DEGs were filtered based on the criteria of logFC > 1 and adj.P.Val < 0.05 (**Figure 3E**), which classified AML patients and healthy donors into two clusters (**Figure 3F**).

### Construction and validation of prognostic model

**Figure 4:**
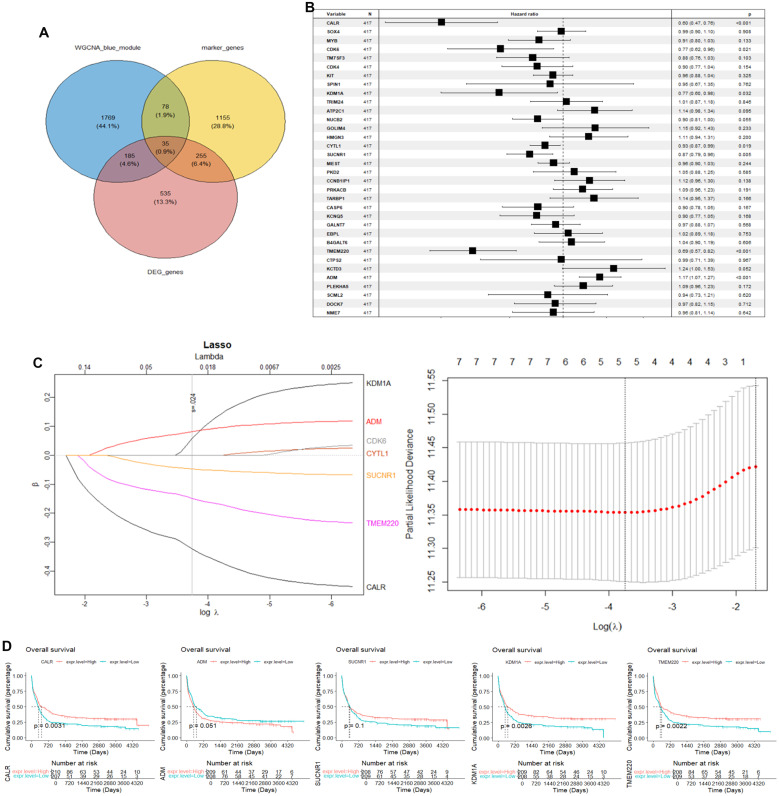
Construction of prognostic signature using GSE37642 dataset. **(A)** Intersection of AML-associated genes, DEGs in key cells, and DEGs between AML and controls. **(B)** Univariate Cox regression analysis of OS. **(C)** LASSO regression of OS-related genes. **(D)** Kaplan–Meier curve of CALR, KDM1A, SUCNR1, TMEM220, and ADM.

**Figure 5:**
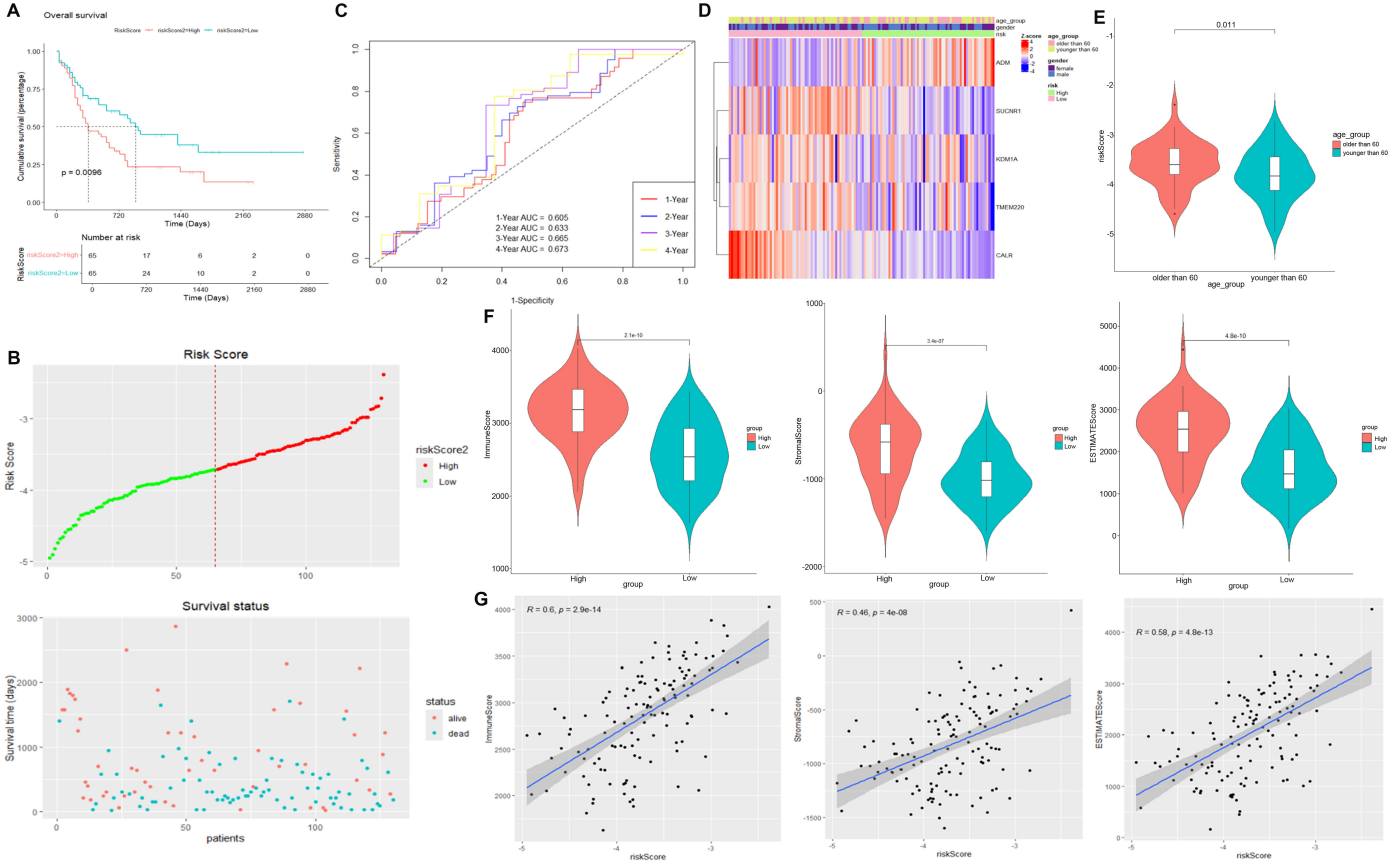
Validation of prognostic signature using TCGA_LAML dataset. **(A)** Kaplan–Meier curve result. **(B)** Risk survival status plot. **(C)** The AUC of the prediction of 1-, 2-, 3-, and 4-year survival rates of AML patients. **(D)** Heatmap of risk model and clinical characteristics. **(E)** Risk score of AML patients older than 60 years and younger than 60 years. **(F)** Scatter plot between immune score, StromalScore, and ESTIMATEScore in high- and low-risk scores. **(G)** Correlation between immune score, StromalScore or ESTIMATEScore, and risk score.

**Figure 6:**
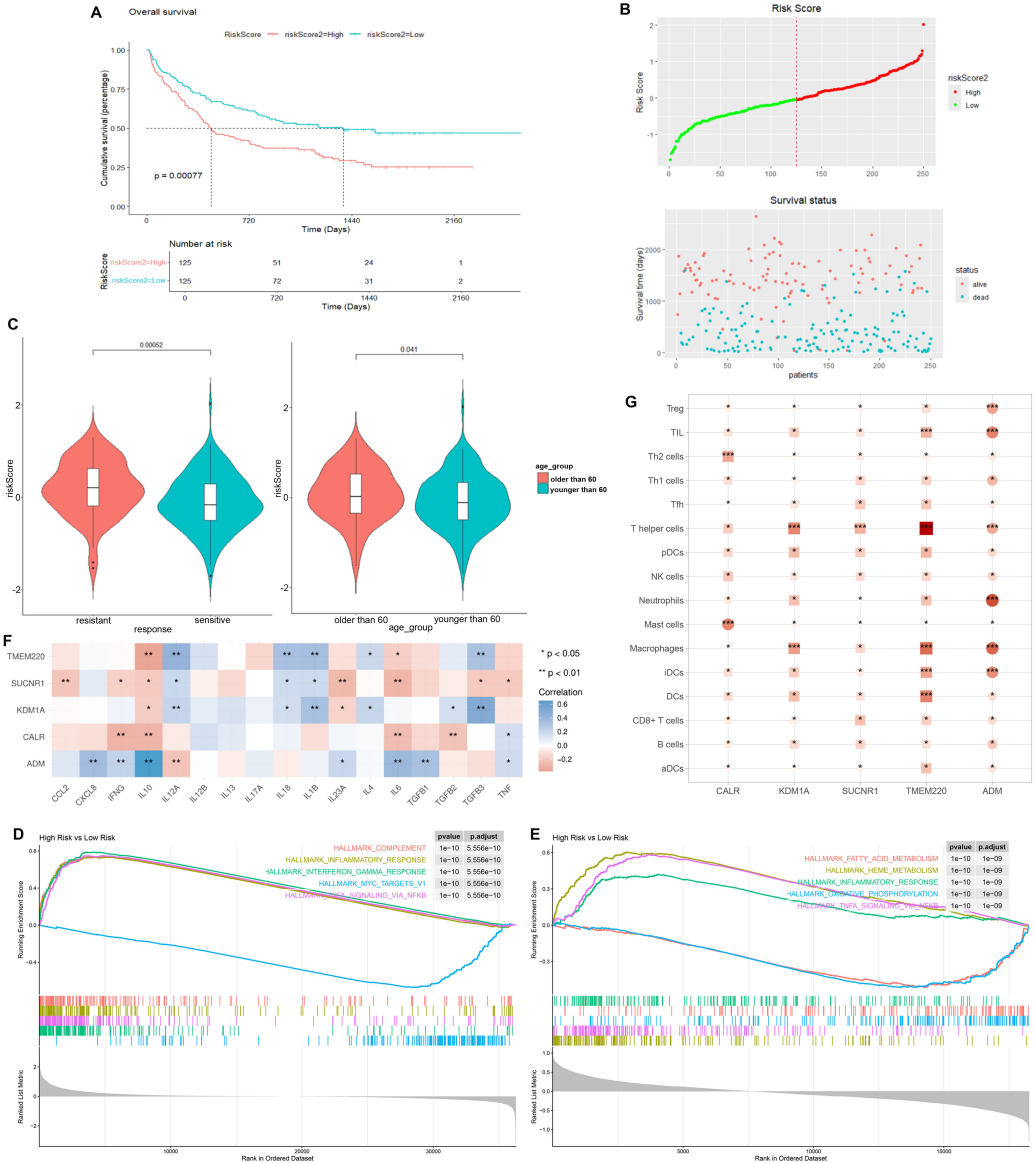
Validation of prognostic signature using GSE106291 dataset. **(A)** Kaplan–Meier curve result. **(B)** Risk survival status plot. **(C)** Risk score of AML patients who were drug resistant and sensitive, and AML patients older than 60 years and younger than 60 years. **(D)** GSEA analysis of risk-model-related genes in TCGA_LAML dataset. **(E)** GSEA analysis of risk-model-related genes in GSE106291 dataset. **(F)** Correlation analysis between five identified signature genes and inflammatory-related using TCGA-LAML dataset. **(G)** Correlation analysis between five identified signature genes and immune cells using TCGA-LAML dataset. *p < 0.05, ** p < 0.01, *** p < 0.001.

We selected the overlapped genes of core cell marker genes, genes in MEblue module, and DEGs between AML patients and controls in GSE114868 dataset as candidates, resulting in 35 genes (**Figure 4A**). Univariate Cox regression analysis was then performed on these candidates in the training set to filter out genes significantly correlated with prognosis, containing CALR, CDK6, KDM1A, CYTL1, SUCNR1, TMEM220, and ADM (**Figure 4B**). Final variables, including CALR, KDM1A, SUCNR1, TMEM220, and ADM, that were used for prognostic model construction were screened using LASSO regression analysis (**Figures 4C, D**). Prognostic risk stratification in AML patients was mathematically derived through weighted integration of multivariate regression coefficients and normalized expression values for five feature-selected genes. The detailed formula is shown below: Risk score = −0.44751 * CALR + 0.28535 * KDM1A + 0.11832 * ADM − 0.20546*TMEM220 − 0.06282 * SUCNR1. Patients were classified into high- and low-risk groups according to the median value of risk score. Further Kaplan–Meier curves analysis suggested that low expression of CALR, SUCNR1, KDM1A, and TMEM220, and high expression of ADM were associated with poor prognosis of AML patients (**Figure 4E**). Moreover, the constructed prognostic model was validated using TCGA_LAML and GSE106291 validation sets. Patients in the TCGA_LAML dataset were categorized into high- and low-risk groups. Kaplan–Meier curves analysis showed that patients with low-risk score have more favorable prognosis than those with high-risk score (**Figures 5A, B**). The ROC curve for overall survival was inferred to further evaluate the availability of prognostic signature, demonstrating that the area under the curve at 1, 2, 3, and 4 years were >0.6 (**Figure 5C**), implying the reliability of the prognostic signature. Available clinicopathological characteristic analysis revealed the significant difference of risk score between patients older than 60 and patients younger than 60 (**Figures 5D, E**). In addition, we observed that patients with high-risk score demonstrate higher ImmuneScore, StromalScore, and ESTIMATEScore than those with low-risk score (**Figure 5F**), and patients’ risk score is positively correlated with ImmuneScore, StromalScore, and ESTIMATEScore (**Figure 5G**). Furthermore, we validated the risk model using the GSE106291 dataset. The results showed the analogous outcome of TCGA_LAML dataset that patients with low-risk score have more favorable prognosis than those with high-risk score (**Figures 6A, B**). Patients who were drug resistant and older than 60 showed higher risk score than those who were drug sensitive and younger than 60, respectively (**Figure 6C**). GSEA analysis of risk-model-related genes in both TCGA_LAML and GSE106291 datasets revealed the significant differences in inflammatory response between high- and low-risk groups (**Figures 6D, E**). We further analyzed the correlation between five identified signature genes and inflammatory-related cytokines using TCGA-LAML dataset, showing that CALR, KDM1A, SUCNR1, and TMEM220 were negatively correlated with IL10 and ADM was positively correlated with IL10. In addition, we observed that CALR, SUCNR1, and TMEM220 were negatively correlated with IL6, and ADM was positively correlated with IL6 (**Figure 6F**). Subsequent analysis demonstrated that all five identified signature genes were significantly correlated with numerous immune cells, indicating the immune correlation of our constructed prognostic model (**Figure 6G**).

### Expression of prognostic-model-related genes in cell clusters

**Figure 7:**
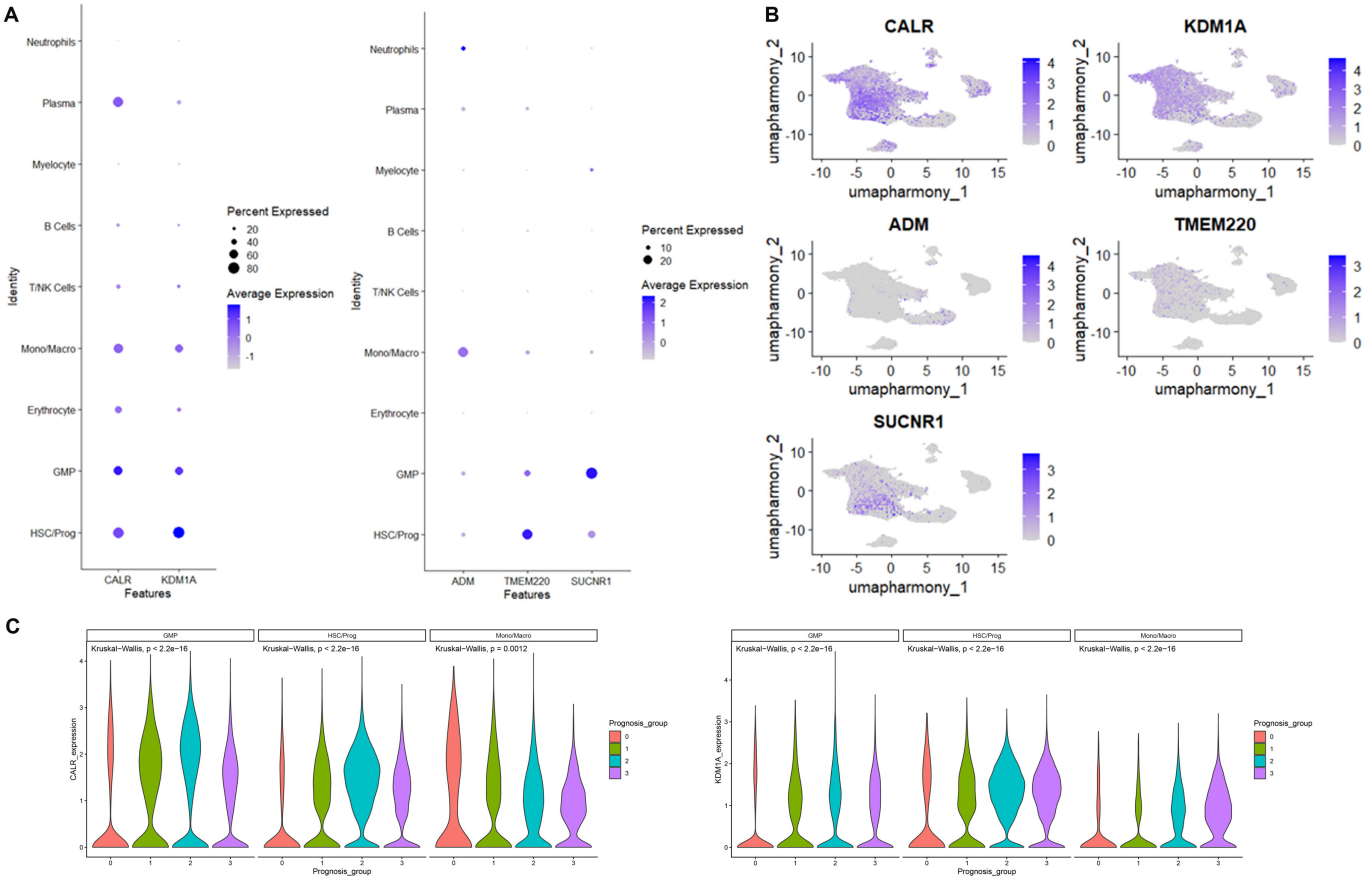
CALR, KDM1A, SUCNR1, TMEM220, and ADM expression distribution in cells identified using scRNA-seq. **(A)** Bubble diagram of CALR, KDM1A, SUCNR1, TMEM220, and ADM expression in cells. **(B)** The umap algorithm was employed to analyze the expression distribution of CALR, KDM1A, SUCNR1, TMEM220, and ADM. **(C)** The expression of CALR and KDM1A in immune cells.

We then went back to the data of scRNA-seq to access the expression of genes in risk model, demonstrating that CALR and KDM1A were primary expressed in Mono/Macro, GMP, and HSC/Prog. SUCNR1, TMEM220, and ADM were primary expressed in GMP, HSC/Prog, and Mono/Macro, respectively (**Figures 7A, B**). The expression of both CALR and KDM1A in Mono/Macro, GMP, and HSC/Prog decreased gradually across patients from group 0 to group 3 (**Figure 7C**).

### Drug sensitivity analysis based on high− and low−risk groups

**Figure 8:**
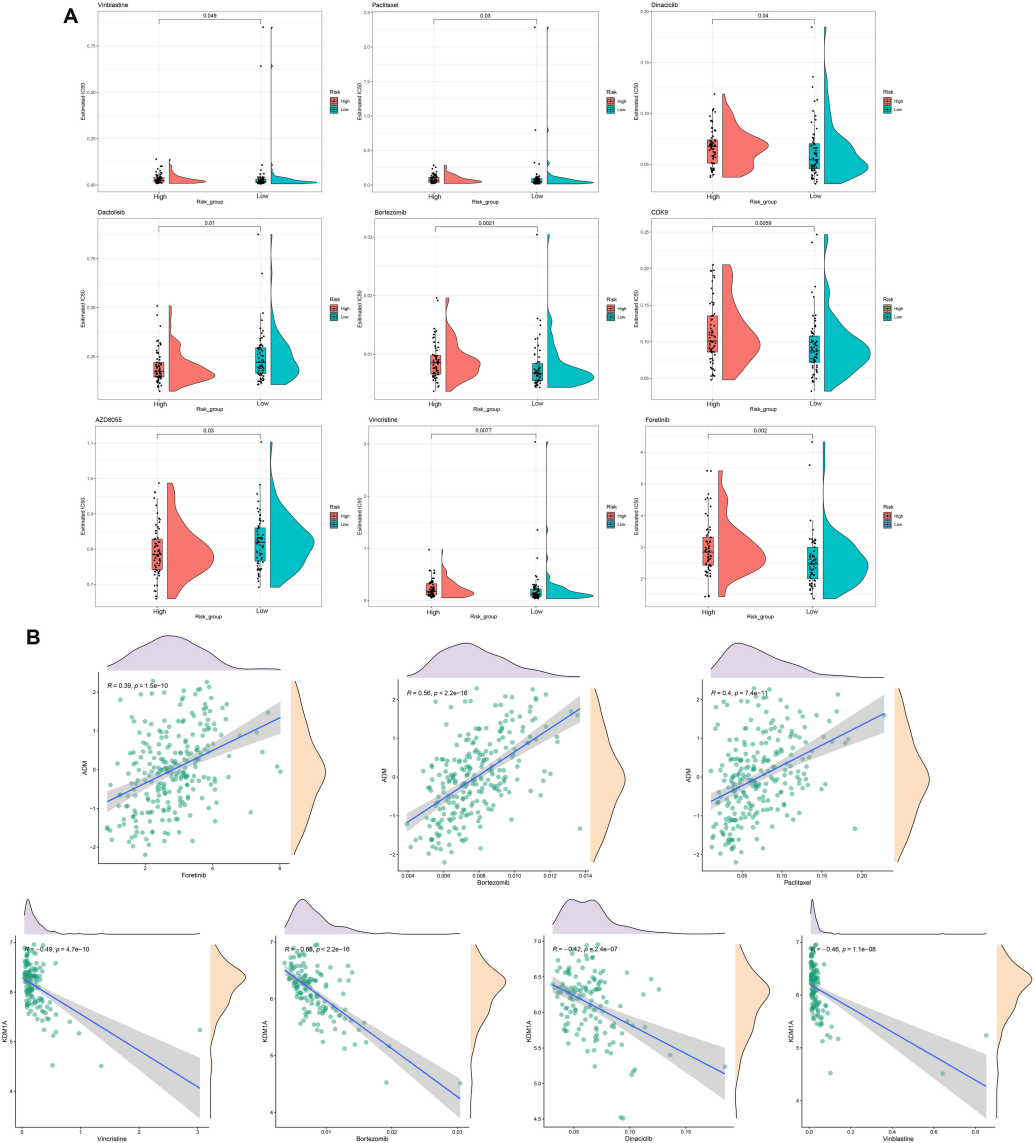
Drug sensitivity analysis. **(A)** Estimated IC50 for different drugs in high- and low-risk groups. **(B)** The correlations between ADM or KDM1A and drugs.

Drug sensitivity analysis of the prognostic signature showed that nine types of drugs represent significant IC50 differences between the high- and low-risk groups, including Vinblastine, Dactolisib, AZD8055, Paclitaxel, Dinaciclib, Bortezomib, CDK9, Vincristine, and Foretinib (**Figure 8A**). Notably, we observed that ADM are positively correlated with Foretinib, Bortezomib, and Paclitaxel, whereas KDM1A are negatively associated with Vincristine, Bortezomib, Dinaciclib, and Vinblastine (**Figure 8B**).

## Discussion

The current study sought to construct a novel risk model for AML patient through comprehensive analysis of internal scRNA data and external bulk RNA data to optimize the precise treatment strategies for patients and improve prognosis. The cells detected using scRNA-seq were classified and annotated into nine clusters, including B cells, erythrocyte, GMP, HSC/Prog, Mono/Macro, myelocyte, neutrophils, plasma, and T/NK cells. The significantly different marker genes of each cell subtype were identified and were subjected to KEGG and GO enrichment. KEGG analysis showed that the maker gene of B cells, myelocyte, and neutrophils were enriched in leukocyte transendothelial migration. In terms of GO enrichment, the marker genes of Mono/Macro, T/NK cells, B cells, myelocyte, and neutrophils were enriched in several immune-response-related terms and leukocyte cell–cell adhesion. Leukocyte transendothelial migration is one of the most key steps in the initiation of an inflammatory immune response, which involves the rapid and transient delivery of preformed soluble elements in the blood to the site of injury, followed by a longer period of leukocyte delivery (12). Because leukocytes cannot swim, they are locally recruited to the site of inflammation through a series of adhesion steps that allow them to attach to the vessel wall, move along the wall to the endothelial border, cross the endothelial and subendothelial basement membranes, and migrate through interstitial tissues (13, 14). A previous study has suggested that inflammation is associated with development from myelodysplastic syndrome to AML (15). It has been considered as a global pathogenic factor in AML, which may involve in various aspects of AML, such as myelosuppression, chemoresistance, and disease progression (16). Inflammation that plays a vital function in the shaping of bone marrow microenvironment is a fundamental constituent of leukemogenesis in AML (17). An inflammation-related gene signature represented by Lasry et al. holds promise for defining risk stratification of AML patients (18). Collectively, our findings suggest the important role of inflammation immune response in the pathogenesis of AML, and the leukocyte transendothelial migration and adhesion in the process of inflammation should be noticed.

ssGSEA scores calculated showed that the scores of all the identified cell clusters between AML patients and control individuals are significantly different, especially GMP, HSC/Prog, Mono/Macro, and myelocyte, which are obviously downregulated in AML patients. Thus, these four cell types were regarded as core cells for subsequent analysis, which contains 1,594 marker genes. Furthermore, we identified AML-associated genes (2,067 genes) and DEGs (1,010 genes) between AML patients and controls using GSE114868 dataset. The overlapping genes of nuclear marker genes, AML-associated genes, and DEGs between AML patients and controls were selected as candidates. The final variables that were used for prognostic model construction were screened in the training set using univariate Cox regression analysis and LASSO regression analysis, including CALR, KDM1A, SUCNR1, TMEM220, and ADM. CALR is a highly conserved chaperone protein that resides primarily in the endoplasmic reticulum and is associated with various biological processes, among them, tumor calcification (19), cell adhesion (20), and immune response (21). Numerous studies have demonstrated the value of CALR in cancer prognostic evaluation. A risk model reported by Chen et. al., which contains CALR, IFNB1, and IFNG, showed a powerful function in bladder urothelial carcinoma prognosis and immune landscape determination (22). Fucikova et al. described that CALR exposure promotes the initiation of antitumor immunity in patients with AML, and it is a reliable prognostic biomarker for AML patients (23). KDM1A is a type of histone lysine demethylase, which has already been found to be associated with a variety of biological processes, such as epithelial–mesenchymal transformation (24, 25) and inflammatory response (26). KDM1A dysfunction was linked to the progression of AML, and inhibitors of KDM1A showed promising therapeutic effect in AML patients (27, 28). SUCNR1 is found in the plasma membrane of multiple cells types, and its activation has been associated with energy metabolism (29) and anti-inflammatory responses (30). Studies of TMEM220 are relatively rare. Limited coverage revealed that TMEM220 is downregulated in hepatocellular carcinoma, and several prognostic signatures contrasted by markers including TMEM220 displayed excellent prognostic prediction effect for hepatocellular carcinoma (31–33). ADM belongs to the amylin/calcitonin gene-related peptide super-family, which has been involved in multiple physiological processes, containing cell migration (34), differentiation (35), and apoptosis (36), and angiogenesis (34). ADM can exert local and systemic anti-inflammatory actions through modulating immune system properties and cytokine secretion (37). As reported by Simonetti et al., the secretion of ADM contributed to the maintenance of an inflammatory phenotype in leukemic cell, therefore leading to relapse and drug resistance of AML patients (38). Moreover, we constructed a prognostic signature based on CALR, KDM1A, SUCNR1, TMEM220, and ADM, which demonstrated a remarkable prognostic value. Further GSEA analysis of risk-model-related genes revealed the significant differences in inflammatory response between high- and low-risk groups. Further correlation analysis revealed significant correlations between hub genes and IL6, IL10, and multiple immune cells. IL-6 has been considered as key pro-inflammatory cytokines impacting the function of hematopoietic cells and promoting inflammatory diseases. IL-6 plays a central role in AML progression by directly stimulating leukemogenic processes, reshaping the inflammatory tumor microenvironment, and orchestrating immunosuppressive mechanisms (39, 40). Its levels serve as a prognostic biomarker, while targeting the IL-6 signaling pathway offers novel therapeutic directions for improving AML treatment (41, 42). IL-10, a key immunomodulatory cytokine secreted by activated immune cells, functions as a regulatory checkpoint, exerting negative feedback regulation to maintain immunological homeostasis and suppress excessive inflammatory activation (43). IL-10 drives AML progression through dual mechanisms. On the one hand, IL-10 suppresses innate immune responses by inhibiting macrophages from releasing pro-inflammatory cytokines (e.g., TNF-α, IL-6) and reducing their antigen-presenting capacity (44). In the AML microenvironment, IL-10 increases the proportion of Breg cells, promotes the secretion of immunosuppressive factors (including IL-10 itself and TGF-β), and suppresses effector T-cell function, thereby facilitating tumor immune escape (45). On the other hand, elevated IL-10 levels in the bone marrow microenvironment of AML patients correlate with increased Treg cell infiltration. These Treg cells further sustain the stemness of leukemia stem cells (LSCs) through IL-10 secretion, driving disease progression and poor prognosis (46). IL-10 levels can serve as a prognostic biomarker and are emerging as a critical therapeutic target for novel immunotherapies, such as CAR-T and IDO1 inhibitors (45, 47). Collectively, the hub genes identified in this study may influence AML patient prognosis by modulating inflammatory responses through complex regulatory interactions with IL-6, IL-10, and immune cells.

In conclusion, our current results emphasized the important function of inflammation immune response in the pathogenesis of AML, and the leukocyte transendothelial migration and adhesion in the process of inflammation should be noticed. In addition, we constructed an inflammation-related risk model that can accurately distinguish survival outcomes in AML patients. However, we have only generalized the role of inflammation in the development of AML, and the specific molecular mechanisms need to be further investigated. Additionally, although the prognostic signature was validated using online dataset, further validation with a large, independent patient cohort is warranted to strengthen the reliability of the prognostic model. The development of novel prognostic signatures is critical for enhancing auxiliary diagnosis and optimized management in cancer patients (48–50). In summary, our current study not only complements the existing prognostic evaluation framework for AML but also extends its applicability.

## Data Availability

The raw data supporting the conclusions of this article will be made available by the authors, without undue reservation.
